# Genetic Diversity and Population History of a Critically Endangered Primate, the Northern Muriqui (*Brachyteles hypoxanthus*)

**DOI:** 10.1371/journal.pone.0020722

**Published:** 2011-06-03

**Authors:** Paulo B. Chaves, Clara S. Alvarenga, Carla de B. Possamai, Luiz G. Dias, Jean P. Boubli, Karen B. Strier, Sérgio L. Mendes, Valéria Fagundes

**Affiliations:** 1 Departamento de Ciências Biológicas, Universidade Federal do Espírito Santo, Vitória, Espírito Santo, Brazil; 2 Instituto Biotrópicos de Pesquisa em Vida Silvestre, Diamantina, Minas Gerais, Brazil; 3 Wildlife Conservation Society, Rio de Janeiro, Rio de Janeiro, Brazil; 4 Department of Anthropology, University of Wisconsin, Madison, Wisconsin, United States of America; American Museum of Natural History, United States of America

## Abstract

Social, ecological, and historical processes affect the genetic structure of primate populations, and therefore have key implications for the conservation of endangered species. The northern muriqui (*Brachyteles hypoxanthus*) is a critically endangered New World monkey and a flagship species for the conservation of the Atlantic Forest hotspot. Yet, like other neotropical primates, little is known about its population history and the genetic structure of remnant populations. We analyzed the mitochondrial DNA control region of 152 northern muriquis, or 17.6% of the 864 northern muriquis from 8 of the 12 known extant populations and found no evidence of phylogeographic partitions or past population shrinkage/expansion. Bayesian and classic analyses show that this finding may be attributed to the joint contribution of female-biased dispersal, demographic stability, and a relatively large historic population size. Past population stability is consistent with a central Atlantic Forest Pleistocene refuge. In addition, the best scenario supported by an Approximate Bayesian Computation analysis, significant fixation indices (Φ_ST_ = 0.49, Φ_CT_ = 0.24), and population-specific haplotypes, coupled with the extirpation of intermediate populations, are indicative of a recent geographic structuring of genetic diversity during the Holocene. Genetic diversity is higher in populations living in larger areas (>2,000 hectares), but it is remarkably low in the species overall (*θ* = 0.018). Three populations occurring in protected reserves and one fragmented population inhabiting private lands harbor 22 out of 23 haplotypes, most of which are population-exclusive, and therefore represent patchy repositories of the species' genetic diversity. We suggest that these populations be treated as discrete units for conservation management purposes.

## Introduction

The genetic structure of primate populations is affected by a number of intrinsic and extrinsic factors, including grouping patterns, sex-biased dispersal, dispersal distances, genetic drift, effective population size and past demographic events such as bottlenecks [1]–[3]. Genetic structure may also be altered by processes acting over different spatial and temporal scales, including geological and climatic-driven events such as habitat contractions/expansions [4]. These processes may be accelerated by anthropogenic interference, such as poaching, deforestation, and fragmentation that can enhance the aforementioned natural effects by geographically isolating and reducing the gene pool [5]–[7]. Quick genetic erosion may follow isolation [8] and may result in remnant populations becoming singular repositories of unique or low frequency alleles for many threatened primate species [9]. Thus, any management plan that targets the maintenance of a species’ remaining diversity will benefit from an assessment of the relevant populations’ genetic differentiation [6], [10], [11].

The Brazilian Atlantic Forest is known for its rich biodiversity, which includes 20 endemic primate species (www.iucnredlist.org). The history of the forest is marked by contraction/expansion cycles thought to be caused by climatic oscillations during the Pliocene and Pleistocene, which likely resulted in three distinct biogeographic domains, or Pleistocene refuges, according to analyses of phylogeography and niche modeling [12]–[15], palynology [16]–[18], and endemism [19]. The Central domain (mainly Bahia, Espírito Santo, and Minas Gerais states) is the largest refuge and is characterized by forest stability in the east and short periods of instability in the west.

The muriqui (*Brachyteles* spp.), one of the endemic primates found in the Atlantic Forest [20], is classified in the Atelinae subfamily (Primates, Platyrrhini) along with three other arboreal genera found exclusively on the Amazon and Central America (*Ateles*, *Lagothrix*, and *Oreonax*). The genus *Brachyteles* includes probably the largest living neotropical primate, which reach over 1.0 m in body length [21], [22]. Initially considered to be monotypic, two species are now recognized: *B. hypoxanthus*, the northern muriqui, and *B. arachnoides,* the southern muriqui [23], [24].

The Atlantic Forest is known as the fourth ranked global biodiversity hotspot [25]. Because of recent deforestation, it now covers less than 8% of its original range (www.sosmatatlantica.org.br). This recent disturbance has had inevitable consequences for the biome’s fauna and flora [26]. Aguirre [20] suggested that about 500 years ago, prior to the arrival of the Europeans in Brazil, 400,000 muriquis inhabited the central-south section of the Atlantic Forest, but this may be an underestimate based on current densities and the species’ range (S.L. Mendes & L. Centoducatte, unpublished data). Strikingly, the most recent species-wide estimate counted only 864 northern muriquis living in 12 isolated populations (varying from 3 to 226 individuals) in small to medium-sized forest patches (44 to 50,000 ha) overlapping the central Atlantic Forest domain in the states of Espírito Santo (ES) and Minas Gerais (MG) [27]. Despite the population reduction in the last 500 years, the current distribution of the species still largely overlaps with what is recognized as the Atlantic Forest central domain.

Social structure, sex-biased dispersal, fission-fusion patterns, group and population sizes are known to contribute to different patterns of genetic structure in social mammals [28], [29]. Northern muriquis live in multimale-multifemale promiscuous groups that may contain more than 100 members. Females disperse from their natal groups at a mean age of six years of age, before the onset of puberty, whereas males are patrilocal [30]. Like in chimpanzees and other social mammals in which females disperse, the expected pattern in muriquis is a homogenous distribution of mtDNA variation within and among social groups. In the absence of geographic barriers, variance among populations will also be reduced although isolation by distance (IBD) may counteract female dispersal [31]. Muriquis also have slow life history traits such as late age at first reproduction (typically nine years of age), long interbirth intervals (three years), and overlapping generations [32]. Moreover, populations of different species inhabiting smaller areas tend to show reduced genetic diversity [33]. All these characteristics make small muriqui demes more vulnerable to allelic fixation than primates with faster life histories or with relatively slow life histories that live in larger populations [34] or larger areas.

Genetic diversity is key for a species to persist and maintain evolutionary potential in the long run [33]. Despite the recent federal enactment of an action plan to reduce the eminent risk of extinction of northern muriquis, little is known about the distribution of genetic diversity within and among populations. Here we combined available demographic and behavioral information with mtDNA diversity of the northern muriqui to assess the effects of habitat disturbance, social organization, and historical demography on the distribution of genetic variation in this critically endangered primate [35]. We predicted that if historic population sizes were large, female dispersal was ubiquitous, and the central Atlantic Forest remained stable, we should find weak geographic structure on the mtDNA. In doing so, we aim to contribute to the species' conservation by describing the spatial distribution of genetic variation and how it might inform management decisions.

## Methods

### Sampling and laboratory procedures

**Figure 1:**
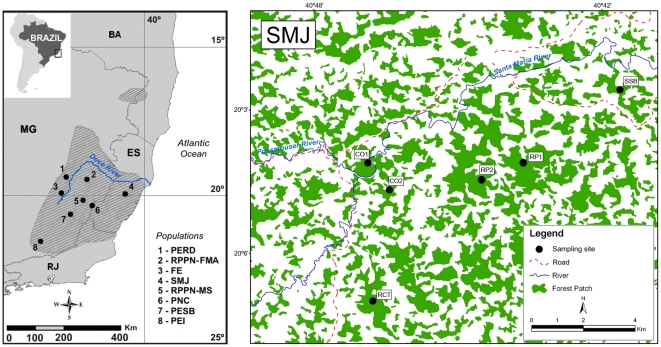
Sampling sites and geographic distribution of the northern muriqui. Map highlighting the southeast section of Brazil with sampled populations overlaid on the northern muriqui distribution, which is based on [36] (left, state acronyms: BA – Bahia, MG – Minas Gerais, ES – Espírito Santo, RJ – Rio de Janeiro). Sampling sites and some of the landscape features at SMJ are shown on the large map (right).

Fecal samples were collected noninvasively between July 2002 and December 2004 from 151 free-ranging animals in eight geographic sites (Fig. 1, Table 1). Each geographic site is treated as population hereafter. These samples represent 17.6% of the total known northern muriqui census population size and cover most of the species’ present range, with the exception of its northern limit [27]. Experienced observers collected fresh droppings from individuals identified by natural fur and facial markings during routine censusing. Animals were neither manipulated nor disturbed during the collecting procedures. The samples were dry-preserved at −20°C in 50 ml vials containing silica beads. We included one additional sequence (GenBank accession number AF213966) [36] from Fazenda Esmeralda (FE) for a total sample size of 152.

**Table 1:** Sampled localities, number of sampled individuals, and molecular diversity indices (*h*, haplotype diversity; π, nucleotide diversity; *s*, number of polymorphic sites).

Locality (State)^1^	Area size (ha)	Minimum population^2^	Sampled individuals	*h* 3	π^3^	*s* 3	UniqueSubstitutions^3^	Haplotype number	Uniquehaplotypes
PERD (MG)	35976	124	23	0.818	0.011	14	3	8	5
PNC (ES-MG)	31853	82	2	-	-	-	-	2	0
PESB (MG)	13210	226	17	0.846	0.013	12	1	7	5
SMJ (ES)	+2000*	84	32	0.754	0.011	13	2	7	5
PEI (MG)	1488	7	1	-	-	-	-	1	1
RPPN-FMA (MG)	957	226	64	0.626	0.010	7	4	3	3
RPPN-MS (MG)	180	41	11	0.000	0.000	0	0	1	0
FE (MG)	44	3	2^3^	-	-	-	-	1	0
Overall	-	793	152	0.905	0.014	21	-	23	-

1: ES: Espírito Santo; MG: Minas Gerais; PERD: Parque Estadual do Rio Doce, PNC: Parque Nacional do Caparaó, PESB: Parque Estadual da Serra do Brigadeiro, SMJ: Santa Maria do Jetibá, PEI: Parque Estadual do Ibitipoca, RPPN-FMA: Reserva do Patrimônio Natural Feliciano Miguel Abdala, RPPN-MS: Reserva do Patrimônio Natural Mata do Sossego, FE: Fazenda Esmeralda.

2: data from October 2005 (27).

3: one sequence from GenBank, accession no. AF213966 [36].

*Estimated from a total area of 13 partially isolated fragments. 3: Calculated only for populations with more than 10 individuals sampled.

Two of these populations are composed of multiple social groups and some of these groups have been the subjects of long-term studies (RPPN Feliciano Miguel Abdala, or RPPN-FMA hereafter, and Santa Maria de Jetibá, SMJ hereafter). Whenever information on groups was available, and the sampling of groups was adequate, we were able to evaluate and compare the genetic differentiation among them (Table S1).

Total genomic DNA was extracted using the QIAamp DNA Stool Mini Kit (Qiagen) following the manufacturer’s protocol. PCR amplification using the species-specific primers Mono1 and Mono2 produced an internal section of 366 bp that bears the first hypervariable segment (HVSI) of mtDNA as previously described [37]. The PCR products were purified with the Wizard SV Gel and PCR Clean-Up System (Promega) and then quantified using 2.0% agarose gels with Low DNA Mass Ladder (Invitrogen). Direct sequencing of one or both DNA strands was performed using the Big Dye Terminator Cycle Sequencing kit and resolved in an ABI Prism 3700 Automated Sequencer (Applied Biosystems).

### Data analyses

#### Genetic diversity

The sequences were aligned using CLUSTAL W [38] and visually inspected in BIOEDIT 7.0.5.3 [39]. A BLAST search [40] confirmed the absence of contamination with exogenous DNA. The *B. hypoxanthus* sequence from GenBank (AF213966) was used as a reference for alignment and haplotype determination. Haplotype determination and population comparisons were performed in ARLEQUIN 3.01 [41]. Genetic variability was estimated by means of haplotype diversity (*h*) and nucleotide diversity (π) [42]. The most appropriate model of nucleotide substitution was selected in jMODELTEST [43] with the Akaike Information Criterion [44] (HKY+I, base frequencies A = 0.3721, C = 0.2469, G = 0.1286, T = 0.2524, proportion of invariable sites of 0.9017, and transition/transversion ratio of 33). Whenever a model was required in subsequent analyses, either the best-fit model or the best approximation of it was used.

Independent of current approximations of population sizes, we examined whether the number of haplotypes observed in large (2,000 ha or larger) *versus* small habitat patches was significantly different. We first inferred accumulation curves of haplotype richness for two sets of populations that were represented by at least 10 individuals. The first group was comprised of the populations found in areas larger than 2,000 ha (PERD, PESB, and SMJ). The SMJ population is comprised of small forest fragments interconnected with the total size of the area exceeding 2,000 ha (Fig. 1). The second group included the two populations found in areas of less than 1,000 ha (RPPN-FMA and RPPN-MS). To construct accumulation curves we generated 20 random samples of 10 individuals each for both of these two groups. Using these samples as input, we ran a rarefaction analysis in ESTIMATES 8.0 [45] with seven cumulative sampling events of ten samples each and 1,000 randomization steps. The final seven samples, each consisting of ten individuals, summed to a simulated sampling effort of 70 individuals, which is approximately the largest combined N for each of these groups (first group N = 72, second group N = 75). The mean and standard deviations of the sampling intervals were plotted against the number of haplotypes with increasing sample size. A paired *t*-test was subsequently used to compare haplotype richness between these groups following the method employed by Peters *et al*. [46].

#### Genetic structure

Analysis of Molecular Variance (AMOVA) was implemented in ARLEQUIN 3.01 using the model of Tamura and Nei [47] to examine the distribution of genetic variance. AMOVA generates variance components and fixation indices that account for variation among populations (Φ_CT_), among social groups within populations (Φ_SC_), and within social groups among populations (Φ_ST_). Statistical significance was assessed with 16,000 permutation steps. Populations represented by a small number of samples (PNC, FE and PEI) were excluded from this analysis. Because an exploratory phylogenetic analysis (not shown) indicated that there were no population sub-aggregations, the AMOVA included no division of groups of populations (the least inclusive hierarchy was the social group, and the most inclusive was the population).

Because the RPPN-FMA and SMJ populations were sampled across different social groups, we tested whether both their overall and within-group haplotype compositions were randomly distributed. First, we compared the haplotype frequencies in the overall sample with a chi-square test. We assumed an extrinsic expectation in which all haplotypes should be found in equal frequencies proportionate to sample size (i.e. expected haplotype frequency  =  N haplotypes/N samples). To control for small sample size we first generated 10,000 random replicates from the sample frequencies (N = 64 for RPPN-FMA and N = 32 for SMJ) and then recalculated the chi-square for each replicate. The proportion of replicates with chi-square values greater than or equal to the observed value is the *P*-value of the test. We repeated this analysis for each social group assuming the expected frequencies of each haplotype to be equal to the observed population frequencies. In addition, to evaluate the contribution of group structure to the distribution of genetic variation we ran 16 AMOVAs, as described above, between these two populations. We removed one social group from each population at a time to detect groups that maximized the Φ_SC_. We used the serial goodness-of-fit plus method [48] to correct for multiple testing (http://webs.uvigo.es/acraaj/SGoFMethod.htm).

To evaluate whether social groups within the SMJ and RPPN-FMA populations are homogenous as a result of female dispersal, we used pairwise Φ_ST_ [49] as a proxy for comparisons of between-group gene flow (see below). In addition to the four social groups previously defined for the RPPN-FMA population [30], we distinguished four social groups for the SMJ population by lumping individuals of different areas based on the groups’ proximity and the connectivity among forest patches (Fig. 1). The four SMJ groups defined were SSB, RP1/RP2, CO1/CO2, and RCT.

Genetic differentiation due to isolation by distance was tested using the Mantel test [50], [51], which measures the relationship between genetic (pairwise Φ_ST_) and geographic distances (Km) as implemented in IBDWS 3.15 [52]. Statistical significance was calculated at 95% confidence interval (CI) with 10,000 randomization steps. We also ran the reduced major axis (RMA) regression analysis in IBDWS to graph the relationship between geographic and genetic distances. We inferred a haplotype network using the median-joining algorithm [53] from NETWORK 4.2.0.1 (http://www.fluxus-engineering.com/sharenet.htm) in order to depict haplotype relationships and explore the association of haplotype groups with geography.

#### Demographic history

**Figure 2:**
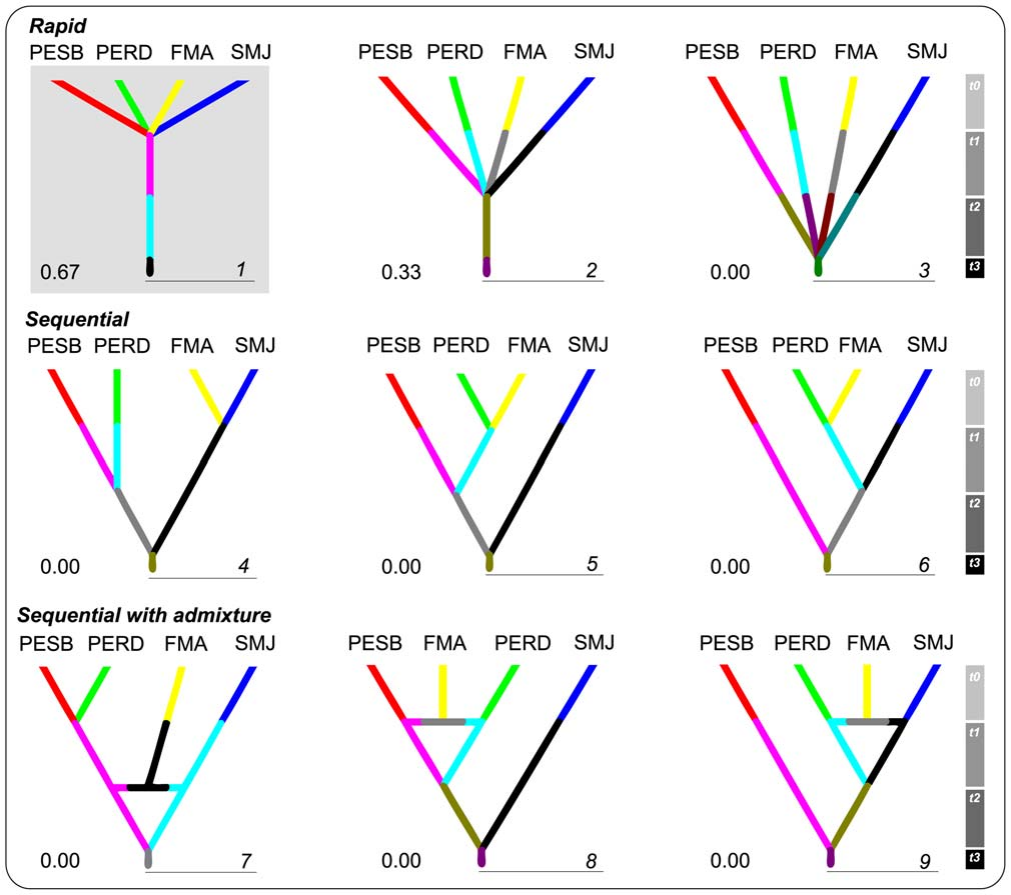
Nine alternative scenarios tested with the Approximates Bayesian Computation approach. Only the four best sampled populations were included (PESB, PERD, RPPN-FMA, and SMJ). The scenarios are organized in three groups of three each. The first group includes scenarios that depicts a rapid or star-like divergence event among the populations (1, 2, and 3). Scenario 1 assumes that one large population split into four populations during the Holocene (t1). Scenarios 2 and 3 push the splitting event further back in time (during the last glacial maximum, t2, and earlier, t3, respectively). The second group includes three scenarios (3, 4, and 5) representing sequential or step-wise divergence among the populations in different times. The third group includes scenarios with sequential divergences and assumes that the population RPPN-FMA (FMA) diverged after an admixture event. Different branch colors represent putative changes in effective population sizes (see Figure S1 for prior set up). The posterior probability of each scenario is shown on the lower left-hand side of its respective diagram.

We adopted a four-step approach to infer changes in the population size of northern muriquis. First, we calculated the neutrality tests *F_S_* [54], *D^F^* and *F* [55], and *R_2_* [56], in DNASP 5.0 [57]. These tests are suitable for detecting different types of demographic events (i.e. sudden expansion, sudden contraction, and bottleneck) in our data set [58]. Very low and significant results of neutrality tests in mtDNA can be interpreted as evidence for population expansion. Significance was calculated after 50,000 coalescent simulations. Second, we calculated the exponential growth rate (*g*) in a coalescent framework using the Bayesian module in LAMARC 2.1.3 [59]. One long Markov Chain Monte Carlo (MCMC) run was performed with 2 million generations, sampling every 20th step and a heating scheme of 4 simultaneous chains with different temperatures (1, 1.2, 1.5, and 3). Third, we used BEAST 1.4.8 [60] to estimate the Bayesian skyline plot (BSP) to depict changes in effective population size over time. A normal distribution from the substitution rate estimated for *Ateles* (see *Substitution rate*) was used in BEAST. We ran the program for 100 million generations, with a sampling interval of 5,000 generations. Finally, we tested nine alternative scenarios for the diversification of four muriqui populations using the Approximate Bayesian Computation (ABC) approach implemented in DIYABC 1.0.4.38beta [61]. We ran a total of 9 million simulations (1,000,000 per scenario) and used the logistic regression method to calculate the posterior probabilities of each scenario (see Fig. 2 and Figure S1 for details). To minimize computation time and circumvent possible sampling bias that would affect the estimates, all coalescent analyses were carried out with a subset of 106 sequences. This smaller sample size was obtained after drawing 25 random samples from each RPPN-FMA and SMJ. Given the shallow haplotype network and lack of strong monophyletic subgroups (see Results), all samples were treated as a single population in the first three treatments.

The long-term female effective population size (*N_ef_*) was estimated using the formula *N_ef_ = θ/2μ*, where *μ* is the substitution rate per site per generation and *θ* is the mutation-scaled effective population size. *θ* was estimated with the coalescent-based methods implemented both in LAMARC and MIGRATE-N 3.2.1 [62], [63], which utilized the maximum likelihood and Bayesian modules, respectively. The LAMARC analysis consisted of three independent MCMC runs of 10 initial chains of 30,000 steps each and 2 final chains of 2 million steps each sampling every 20^th^ step. In MIGRATE, we ran 50 replicates of one long chain containing 10,000 samples each, sampling every 500^th^ step. The prior distribution of *θ* was set as exponential with window (min. 0.0, mean 0.01, max. 0.1, delta 0.01). This analysis had a static heating strategy to explore the parameter space more effectively (swapping interval  = 1; four chains with start temperatures  = 1, 1.5, 3.0, and 1,000,000). The initial 10% genealogies were discarded as burn-in in all coalescent runs. Convergence for the coalescent analyses was assessed by inspecting the effective sample sizes and the stationarity of posterior distributions in TRACER 1.4.1 [64] or the R statistical package [65].

#### Substitution rate

Fossil and molecular data suggest that the New World monkeys split from Old World monkeys roughly 35-65 mybp [66]–[68]. Because they faced different evolutionary pressures since this time, substitution rates between these major lineages may differ, which would influence estimations of effective population size. Moreover, mtDNA substitution rates have been shown to correlate with basal metabolic rate, generation length and longevity in mammals [69], implying that using Old World monkeys’ rates in atelids may be misleading. We approximated a substitution rate (*μ*) more suitable for atelids using the *Ateles* HVSI sequences, the topology, the split dates from Collins & Dubach [70], and a substitution model estimated in jMODELTEST. The sequences were trimmed to overlap the 366-bp segment used in this study. To attach uncertainty to the dates estimated by Collins & Dubach [70], two normally-distributed calibration points were used, one for the time of the most recent common ancestor (TMRCA) on the root of the tree (3.59 mybp, 95 CI ±1.5 mybp), and the other for the split between the South and Central American *A. geoffroyi* subspecies (2.0 mybp, 95 CI ±1.0 mybp). The probable maximum age of this event is likely to coincide with the closure of the Panama Isthmus about 2.8 mybp [71]. An exploratory BEAST run confirmed that the molecular clock assumption could not be rejected for this data set. We then ran BEAST with the HKY+I+G model for 30 million generations and sampled every 3,000th generation. A uniform distribution was assigned to the substitution rate parameter to allow the program to explore a broad interval of values that varied from 0.05×10^−7^ to 5.5×10^−7^ substitutions/site/year (s/s/y). Multiple runs incorporating the recommended adjustments from the output were carried out to check for convergence. The outputs were inspected in TRACER.

## Results

### Sequence quality and variation

It is likely that an orthologous mtDNA copy was sequenced instead of exogenous contaminants or nuclear insertions because: (i) we used a species-specific pair of primers that successively failed to amplify high-quality human DNA; (ii) we detected one single PCR amplicon of the expected size in all individuals; and (iii) no extremely variant sequences were detected. Moreover, fecal samples are richer in mtDNA molecules than they are in nuclear DNA, and the natural degradation of fecal DNA makes nuclear copies even scarcer, reducing the likelihood of amplifying *numts*. All BLAST searches yielded E-values that were very close to zero with the platyrrhine HVSI sequences available, again reinforcing the lack of contamination.

We successfully aligned the 366-bp HVSI segment from 151 newly sequenced northern muriqui individuals from 8 populations with the additional GenBank sequence from FE. Twenty-tree haplotypes (22 newly described) were resolved based on 21 segregating sites (20 transitions, 1 transversion and no indels). Thirteen sites were parsimony-informative. Haplotype sequences are available in GenBank under accession numbers JF769864-JF769886 (Table S2).

**Figure 3:**
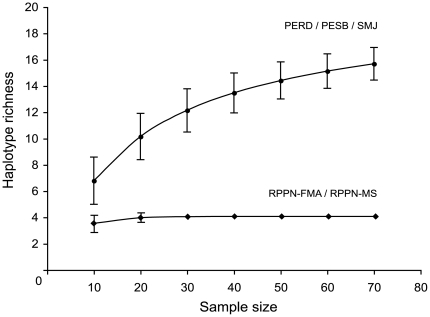
Rarefaction analysis showing the changes in haplotype richness relative to successive increments in sample sizes. Rarefaction curves of two groups of populations sampled for at least ten individuals. The test was significant for the differences in haplotype richness between these groups (*t* = 12.5, *df* = 19, P<0.0001). The mean number of haplotypes found in 10 samples randomly drawn from the group PERD/PESB/SMJ was 7.0 (SD = 1.3), whereas the mean was roughly half as much (*X* = 3.5, SD = 0.2) for the groups found in smaller areas (RPPN-FMA/RPPN-MS).

The overall haplotype diversity was high (*h* = 0.905), and the nucleotide diversity was remarkably low (π = 0.0135). Haplotype h16 was found with the highest frequency in the overall sample set (22%) and was twice as frequent as h17, the second most frequent. Both h16 and 17 were exclusive to RPPN-FMA (Table S1). This distribution likely reflects the joint effects of a sampling bias because the RPPN-FMA encompasses 42% of the total sample and the low number of haplotypes in this population. Within each population, the haplotype and nucleotide diversities were relatively low, with RPPN-MS having only one haplotype and thus diversity measures equal to zero (Table 1). From the populations with at least ten individuals sampled, PERD harbors the highest number of haplotypes (8), and RPPN-MS harbors the lowest (1). Almost all haplotypes (19 out of 23) are exclusively found in only one of the eight populations: PERD, PESB, and SMJ (5 haplotypes each), RPPN-FMA (3) and PEI (1). The first four populations together have all haplotypes, except h5. The h3 haplotype, which is found in PERD, SMJ, and FE samples, is identical to the GenBank reference sequence from the FE locality. The rarefaction analysis showed that populations living in larger areas maintain significantly more haplotypes than the ones that are restricted to areas less than 1,000 ha (Fig. 3), whereas the differences among populations from larger areas themselves seem subtle (Table S1).

### Genetic structure

The AMOVA yielded high fixation indices between populations (Φ_ST_ = 0.49, *P*<0.001) revealing a finer pattern of genetic structure and indicating that only 51% of the total variation can be found within the social groups sampled. At first glance, a significant amount of the genetic variation seems to be partitioned among social groups within each population (Φ_SC_ = 0.33, *P*<0.001). However, when we excluded the group SSB (SMJ), this index dropped substantially and became nonsignificant, (Table S3). The uneven haplotype frequency within this group is supported by the randomization tests, which also showed uneven frequencies within RPPN-FMA and SMJ populations (Table S1). The high and significant fixation index of population subdivision (Φ_CT_ = 0.24, *P*<0.037) could be a consequence of isolation by distance, however the Mantel test (r = 0.12, *P* = 0.67) and RMA analysis (not shown) do not support IBD.

**Figure 4:**
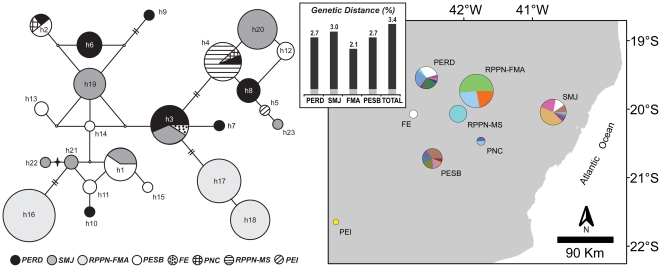
Haplotype network, haplotype distributions, and intrapopulation genetic distances. Median-joining network depicting haplotype relationships is shown on the left side. The areas of the circles are proportional to the relative abundance of each haplotype (the smallest circle represents 1 sample, the largest circle represents 33 samples). Each node between two haplotypes or median vectors (small open circles) accounts for one mutational step (transition or transversion), unless indicated by two vertical dashes, which account for two transitions. A star shows the single transversion detected in the respective node (h21–h22). The map on the right side shows the haplotype frequencies (pie charts) within each population. Each color represents one of the 23 haplotypes. The chart area is scaled to the sample size. The bar graph (inset) in the center of the figure displays the lower and upper bound genetic distances calculated between pairs of haplotypes within each proposed management unit (MU) population. While the lower bound distances were the same in all groups (0.3%, gray section of each bar), intrapopulation divergences (black sections of each bar) did not deviate substantially from the overall (TOTAL) level.

The overall pattern depicted by the haplotype network is rather complex (Fig. 4), also revealed by shallow phylogenetic trees (data not shown). Most haplotypes differ from their nearest neighbor by one mutation, yielding pairwise distances that range between 0.3 and 3.4%, with an overall average of 1.5% (Table S4). Distant populations shared several nucleotide polymorphisms (Table S1, Table S2), resulting in no correlation between haplotypes and geography. It appears that h3 is the deepest diverged haplotype because it is present in 13 individuals from 3 geographically distant populations (FE, PERD and SMJ) [4], [72].

The pairwise Φ_ST_ indicates a higher amount of gene flow within RPPN-FMA’s than SMJ’s groups (Table 2), consistent with field observations and differences in dispersal opportunities seen in the continuous population at the RPPN-FMA but not in the metapopulation at SMJ. Notably, one single haplotype was found in all members of the SSB social group in the SMJ. Because 14 out of the 16 members of SSB were analyzed, it is unlikely that the two additional members have a different haplotype. At the same time, the SSB haplotype was not found in any of the other five areas surveyed.

**Table 2:** Pairwise Φ_ST_ between muriqui social groups for RPPN-FMA (upper left) and Santa Maria do Jetibá (lower right).

	Matão	Jaó	M2	Nadir	SSB	RP1/RP2	CO1/CO2
Matão	-						
Jaó	-0.042	-					
M2	0.223	0.117					
Nadir	0.329*	0.254*	-0.131	-			
RP1/RP2					0.870*	-	
CO1/CO2					0.722*	0.098	-
RCT					0.946*	-0.266	-0.136

*P<0.05.

### Demographic history

**Figure 5:**
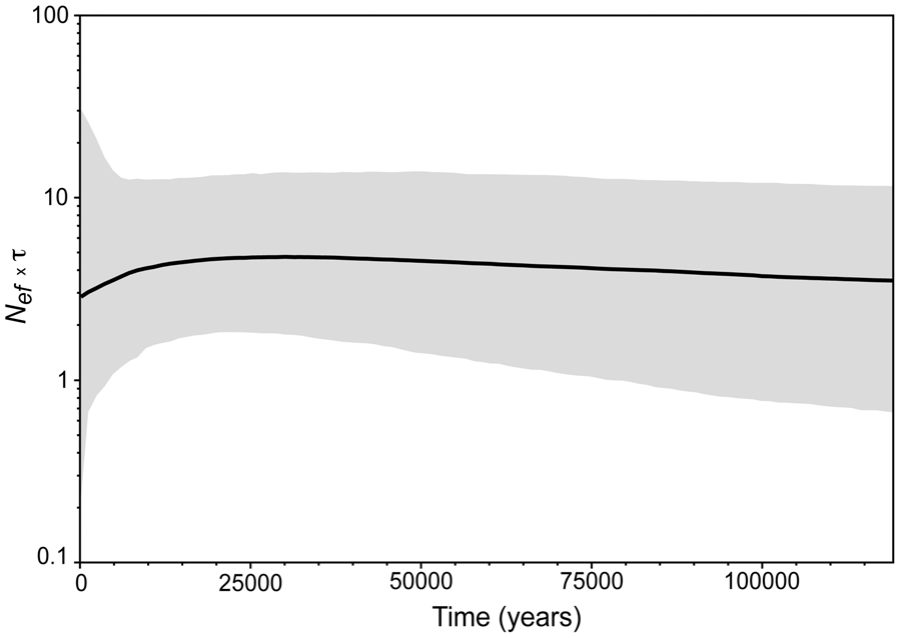
Bayesian skyline plot depicting the population size of northern muriquis over time. Bayesian skyline plot showing an overall stable population size in northern muriquis. A modest population decline near the end of the last glacial maximum (roughly 10000 years ago) may have occurred and it is possibly associated with the recent population subdivision shown in the ABC analysis (see Fig. 2). The solid line is the median, and the shaded area around it is the 95% HPD estimate of the historic female effective population size (*N_ef_*) not corrected for generation time (τ). Timing of events was estimated assuming a substitution rate of 3.7×10^−8^ s/s/y (2.1×10^−8^<*μ*<6.0×10^−8^ s/s/y, see text for details). Time is shown from 0 (present) to 120 kya (the lower estimate of the 95% HPD around tMRCA).

Based on the nonsignificant and near zero results of neutrality tests in the total sample, we could not reject the hypothesis of demographic stability (*F_S_* = −4.08, *D^F^* = 0.0, *F* = 0.0, and *R_2_* = 0.09 all *P*>0.05). Likewise, although the exponential growth rate estimated in LAMARC has a broad confidence interval, the point estimation indicates at most a rather modest growth. We could not reject a nongrowing population scenario with the LAMARC results because the 95% most probable estimate interval includes zero (*g* = 160, −62<*g*<591). The demographic scenario recovered in the BSP (Fig. 5) and the one favored by the ABC (Scenario 1, posterior probability 0.67) were in agreement with the previous analyses, and suggest that there is no mtDNA evidence for dramatic population changes in northern muriquis. The ABC analysis also suggests that the current configuration of isolated populations is a more recent and probably occurred in the Holocene (<10,000 years, Scenario 1, Fig. 2), and most likely not before the last glacial maximum about 20,000 years ago (Scenario 2, posterior probability 0.33).

The coalescent analyses yielded nearly perfect overlap between the LAMARC and MIGRATE estimates of *θ*, though we report only the latter here (median *θ* = 0.01803, 0.0091<*θ*<0.0285 95% CI). The median substitution rate estimated for *Ateles* was *μ* = 3.7×10^−8^ s/s/y (95% HPD, 2.1×10^−8^<*μ*<6.0×10^−8^ s/s/y). Assuming a generation time range of 15–20 years (age at last birth in muriquis is unknown, which makes the calculation of generation time difficult) we estimated a point *N_ef_* = 13,923 females (3,792–45,238 females when the 95% CI for both *μ* and *θ*, and generation interval range were incorporated into the estimation).

## Discussion

### Northern muriqui HVSI diversity

We found high *h* and very low π both in the total samples and within populations. The low π can be seen directly in the mtDNA sequences with few polymorphic sites, which is likely a consequence of relatively low substitution rate in HVSI and the muriqui long generation times. It is notable that in SMJ, RPPN-FMA and RPPN-MS haplotype diversities are comparatively low (i.e. *h*<0.8). While RPPN-FMA and RPPN-MS were extensively sampled relative to their respective population sizes, some less sampled populations exhibited a far greater ratio of haplotypes to sample size. This discrepancy was most evident in PESB (7/17), PERD (8/23), and SMJ (7/32), and suggests that genetic variation is associated with habitat size but not necessarily with current population size, as confirmed by the rarefaction analysis.

Our results agree with previous works showing that the number of social groups is inversely proportional to the rate of loss of genetic variation owing to increased effects of inbreeding [29], [31]. For instance, PESB, PERD and SMJ populations with 7, 12 and 13 groups, respectively [27], [73] have a high number of haplotypes per sample collected. In addition, we believe that more than number of social groups, the history of each population explains the genetic differences. For instance, the RPPN-FMA, with only 3 haplotypes in 63 samples, currently has four social groups, instead of the two social groups it had 28 years ago when the population was much smaller [30].

There are few intraspecific genetic studies of neotropical primates using the HVSI marker in multiple populations, which limits our ability to put our data in a broad comparative context. However, the five available surveys that sampled at least ten individuals (Table S5) showed that mtDNA variation in *B. hypoxanthus* is comparatively low. For instance, after rescaling the HVSI sequences of *Alouatta caraya* to match the segment of northern muriquis, we found 31 haplotypes in half as many samples as the present study, with comparable indices of *h* but lower π (Table S4). It is worth noting, however, that 57 out of the 73 *A. caraya* samples were drawn from one single population [74], which may lead to an underestimation of the overall species diversity. Likewise, Di Fiore [75] found 31 haplotypes in 61 woolly monkeys sampled across 3 different sites in eastern Ecuador. Social groups in this closely related species supported as many as 10 haplotypes, which is similar to the maximum within-group value in muriquis (8). Northern muriqui’s HVSI nucleotide diversity is also consistently lower than that of threatened Old World anthropoids, including the critically endangered snub-nosed monkey [76], and the great apes such as Eastern and Western gorillas, bonobos, Western chimpanzees [77], [78] and Bornean orangutans [79].

In a study of allozyme diversity, Pope [80] reported high levels of heterozygosity (*H = *11%) and high F_ST_ (0.413) in 32 allozyme loci from two *Brachyteles* populations that are currently considered to be representative of the two species (Fazenda Barreiro Rico, Sao Paulo for *B. arachnoides* and Fazenda Esmeralda, Minas Gerais – our FE population – for *B. hypoxanthus*). The results from Pope's analysis suggest that the rapid and recent decline in population size has still not affected the heterozygosity, a result that is corroborated by our high *h* value.

### RPPN-FMA versus SMJ micro-geographic structure

The degree of genetic differentiation among social groups depends on the interaction of processes affecting them when they are first formed and between their onset and their eventual dissolution [81]. Thus, the global degree of genetic subdivision in socially structured species is determined by the effects of sampling and mixing associated with three interrelated behavioral features: mating, dispersal, and the formation of new social groups. Following the initial founding event, social dynamics determine population genetic structure through the interplay of gene flow and genetic drift.

The high field-observed migration rate in RPPN-FMA is in agreement with pairwise Φ_ST_ and the complex pattern of formation and distribution of groups in this population. At the onset of the systematic monitoring in 1982, only two groups, the Matão and Jaó groups, were present. The Matão group grew from its original 22 members, including six adult males and eight adult females, to 77 individuals as of January 2005, and it has never fissioned. The Jaó group, by contrast, underwent its first fission event in 1998, which resulted in the Matão 2 (M2) group and a second fission in 2002, which resulted in the Nadir group and included a male unit that still maintained transient associations with both the Jaó and Nadir groups when the genetic samples used in the present study were collected [30]. Between 1983 and 2004, field observations confirmed that 56 females were exchanged between the four groups in this population [30]. Thus, in the last two decades the average migration rate in the RPPN-FMA population was close to 14 females/group/generation. This is most likely an inflated migration rate because of the strongly female-biased composition of the population during these years, but it illustrates well the fact that a contiguous habitat facilitates female dispersal among social groups [34].

By contrast, SMJ has a total confirmed population size of at least 84 individuals distributed into 13 social groups that occupy small forest fragments from 60 to 350 ha in size. Systematic monitoring of the SMJ population began in 2005, and aerial pictures from the 1970’s and 2002 indicate that fragmentation and deforestation was more severe 40 years ago, when the groups were more isolated than they are today. Dispersal events between SMJ groups are reduced compared to RPPN-FMA groups, which may have contributed to the fixation of haplotype h20 in the SSB group. Although the migration rates estimated with mtDNA should not be regarded as “true” estimates of current gene flow, all indices, and a closer inspection of the raw genetic data, clearly suggest that successful migration among some SMJ groups is either very low when or negligible when compared to RPPN-FMA. The constraints on dispersal in SMJ muriquis are also implied by the sightings of two solitary females on the periphery of their natal groups, where they have been seen engaging in unorthodox interspecific associations with brown howling monkeys (*Alouatta guariba*, S.L. Mendes pers. obs.). Low dispersal rates appear to be associated with isolation by landscape resistance, which includes distance between social groups, forest connectivity, topographic features, and the presence of barriers such as rivers, roads, and pastures in SMJ. They can be also influenced by the small number of females able to disperse.

An intriguing fact is that members of the SSB group, which is only 6 Km away from the nearest group (RP1), do not share haplotypes with other groups in SMJ, and no females have been observed immigrating into this group. Roads or forest fragmentation may play a role in limiting gene flow between this and other muriqui groups in this population. As is common in atelids, muriquis are extremely adapted to suspensory locomotion via brachiation and by using their prehensile tail as fifth limb; they are also known to cross short distances on the ground [82]. Thus, although the dependence on tree canopies may pose a major constraint on dispersal over long distances, we cannot rule out the hypothesis that a founder effect was the major force driving the genetic differentiation of the SSB group.

### Macro-geographical structure and demographic history

The shallow genetic structure observed in muriquis is probably driven by female dispersal and the absence of long-term geographic barriers that could have isolated the populations analyzed in the present study. This is strengthened by the fact that a deeply diverged haplotype (h3) is found in three distant populations (FE, SMJ, and PERD). Haplotype sharing between the SMJ and FE populations, which are in the same latitude and span roughly the widest longitudinal distance between any two populations (∼227 km), and the lack of isolation by distance with the ABC analysis results suggest that gene flow existed along forested routes that are no longer available. Nonetheless, the high AMOVA (Φ) fixation indices indicate that the apportionment of genetic diversity within social groups and populations is higher than would be predicted for a species in which females disperse. This implies that at least some level of past genetic structure, higher levels of within-population admixture, random genetic drift in the more recent past [83], and the presumably contemporaneous extinction of intermediate populations account for the current distribution of genetic variation in muriquis (see below).

Our estimate of historical *effective* population size is consistent with previous reports that indicate a past *census* population size at least one order of magnitude higher than the current size [20]. At the time of our sample collection, roughly 226 muriquis lived in the fairly small (957 ha) forest at the RPPN-FMA [30]. Using known densities, a simulation study suggested that a minimum area of suitable habitat of 11,570 ha would be needed to sustain a population of 700 individuals [84], [85]. Based on these estimates, a total area ranging from 850,000 to 3,305,714 ha would be required to sustain about 200,000 northern muriquis (the historical census size estimated from [20]). This is at most only half the original species range (for reference, the ES state alone spans over a 4,559,700 ha. see Fig. 1). If we extrapolate the point estimation of the female effective population size and assume a long-term 1∶1 ratio of males and females and equal variance in reproductive success among males and females, we obtain a *N_e_* = 27,846 individuals, which seems quite high for a species with such a restricted geographic distribution compared to other primates (e.g. [86], [87]), and yet is only about 10% of the estimated past census size.

It is notable that our *μ* estimation is low compared to other primates. It is at the lower end of the usual phylogenetic substitution rates used in great apes and human HVSI, which often range from 4.7×10^−8^ to 16.5×10^−8^ s/s/y [88]; but it is consistent with estimates of other large-bodied mammals such as whales and carnivores (e.g. [89], [90]). Compared to Old World monkeys of similar size, atelin monkeys have slow life histories that may be associated with phylogenetic effects [91], [92]. We hypothesize that their slow life histories, including greater longevity [69], may account for their low substitution rates estimated in the present study. Although we have incorporated some degree of uncertainty by using flexible calibration points, we caution that our estimated substitution rate is conditional on the divergence times available, which rely on the scarce platyrrhine fossil record. These estimates, however, do not affect the qualitative changes of effective populations sizes; hence our main conclusions are valid even if the substitution rate varies.

Given the evidence from this and other studies [12]–[15], the biogeographic scenario that best fits the mtDNA variation in *B*. *hypoxanthus* should include the following elements: (i) long-term persistence of forest refugia during Pleistocene climate changes; (ii) female-biased dispersal; (iii) the ability to disperse over significant distances relative to the species’ distribution when the habitat is continuous; and (iv) relatively large effective population size. The recent elimination of intermediate populations because of habitat loss may have created the current lack of clear phylogeographic partitioning even though there are significant differences in haplotype frequencies among populations (including several exclusive haplotypes). We acknowledge, however, that these conclusions are based on a single locus and alternative explanations would also be possible if the mtDNA has been influenced by idiosyncratic processes (e.g. selective sweeps) [93]. Moreover, isolated populations to the northern limit of the distribution may show a higher degree of genetic differentiation.

### Implications for conservation

To our knowledge, *Brachyteles hypoxanthus* has the lowest HVSI diversity hitherto reported for a neotropical primate (Table S5), and likely among the lowest known for threatened primates. The distribution of its diversity seems to be influenced by recent habitat fragmentation, population decline, and the species demographic history. This diversity is now scattered across remnant populations with some less well-protected populations retaining a significant component of the remaining variation (e.g. SMJ). Moreover, because haplotypes from the subdivided populations in our sample differ significantly in frequency but do not show a reciprocally monophyletic relationship or a clear pattern of geographic structure, we suggest that the PERD, PESB, RPPN-FMA, and SMJ populations be prioritized as discrete management units (*sensu* Moritz [94]). Together, these populations represent 75% of the entire known census population size and they contain 22 of the 23 identified haplotypes. Their representation of the genetic diversity of the species makes them top conservation priorities.

Because the population sizes are below what is widely accepted as minimum viable population sizes thresholds [95], the northern muriqui will remain a conservation-reliant species, requiring active management for generations to come [96]. To reduce inbreeding effects, translocation of females between fragments is already under way in some populations. And because no deep phylogeographic partitions are observed in northern muriquis, most populations seem to be a small part of what resembles a historical quasi-panmictic population, which offers a relatively less complex scenario for managing translocations. Exogamic effects are less likely to affect these populations because they have not been evolving independently under different selective pressures for an extended time [97]. Nonetheless, additional markers (e.g. microsatellites) and more extensive sampling of some populations will be required to design more effective conservation guidelines. Translocations should be carefully evaluated on case-by-case basis in order to take the complexities of each one into account.

The ultimate goal of the National Muriqui Action Plan [98] with regard to the northern muriqui is to effectively change the species status from “critically endangered” to “endangered” by 2020. It also recognizes the importance of understanding how the genetic diversity is distributed among the remnant populations to aid in delineating holistic management actions. Despite the limitations of a single-locus appraisal, our study spotlights the need of protecting key populations that sustain high levels of genetic diversity, identifying ways to reestablish gene flow between neighboring populations and isolated social groups, and directing conservation efforts to populations other than those already fully protected. Assessing the genetic characteristics of unsustainable populations before they vanish will serve to inform us the extent to which northern muriquis will lose part of their evolutionary heritage, and what can be done to mitigate the effects of this loss on the species survival.

## Supporting Information

Figure S1 Prior/Scenario settings used in the Approximate Bayesian Computation Analysis.
(PDF)

Table S1 Absolute and relative frequencies of haplotypes per population (and group, if known).
(DOC)

Table S2 Polymorphic sites for the HSVI segment in the northern muriqui.
(DOC)

Table S3 Series of 16 AMOVAs excluding one group from both RPPN-FMA and SMJ at a time.
(DOC)

Table S4 Tamura-Nei pairwise genetic distances between haplotypes.

Table S5 Summary table of genetic diversity parameters of HVSI sequences in northern muriquis and available neotropical primates.
(DOC)
