# Correction: Genetic Diversity and Population History of a Critically Endangered Primate, the Northern Muriqui (*Brachyteles hypoxanthus*)

**DOI:** 10.1371/annotation/1bdb2ee6-ceb8-4b3a-9773-e9a82cf22688

**Published:** 2011-07-21

**Authors:** Paulo B. Chaves, Clara S. Alvarenga, Carla de B. Possamai, Luiz G. Dias, Jean P. Boubli, Karen B. Strier, Sérgio L. Mendes, Valéria Fagundes

This work was sponsored by Ministério do Meio Ambiente "PROBIO - MMA/BIRD/GEF/CNPq" (http://www.mma.gov.br, JPB, LGD, VF, and SLM), Conselho Nacional de Desenvolvimento Científico e Tecnológico (http://www.cnpq.br, SLM and VF), Critical Ecosystem Partnership Fund (http://www.cepf.net) and Fundação de Amparo à Pesquisa do Espírito Santo (http://www.fapes.es.gov.br, VF), and the Zoological Society of San Diego (http://www.sandiegozoo.org, JPB). KBS has supported the long-term demographic and behavioral studies at RPPN-FMA with funds from NSF (http://www.nsf.org, BNS 8305322, BCS 8619442, BCS 8958298, BCS 9414129, BCS 0621788, BCS 0921013), the National Geographic Society (http://www.nationalgeographic.com), the Liz Claiborne and Art Ortenberg Foundation (http://www.lcaof.org), Fulbright Foundation (http://www.fulbright.org), Sigma Xi Grants-in-Aid (http://www.sigmaxi.org), Grant 0000213 from the Joseph Henry Fund of the NAS (http://www.nationalacademies.org), WWF (http://www.wwf.org), L.S.B. Leakey Foundation (http://www.leakeyfoundation.org), Chicago Zoological Society (http://www.brookfieldzoo.org), Lincoln Park Zoo Neotropic Fund (http://www.lpzoo.org), Margot Marsh Biodiversity Foundation, Conservation International (http://www.conservation.org), and the Graduate School of the University of Wisconsin-Madison (http://www.grad.wisc.edu). PBC and CSA were supported by CNPq and Petrobras (http://www.petrobras.com.br) scholarships, respectively. The funders had no role in study design, data collection and analysis, decision to publish, or preparation of the manuscript. 

