# Supplementary material for: Genetic Diversity and Population History of a Critically Endangered Primate, the Northern Muriqui (Brachyteles hypoxanthus)
Source: PLoS One. 2011 Jun 3;6(6):e20722. doi: 10.1371/journal.pone.0020722 (PMC3108597; doi:10.1371/journal.pone.0020722)
Supplement: Figure S1 — Prior/Scenario settings used in the Approximate Bayesian Computation Analysis. (PDF) [file pone.0020722.s001.pdf]

Figure S1: Prior/Scenario settings used in the Approximate Bayesian Computation analysis.

Rapid

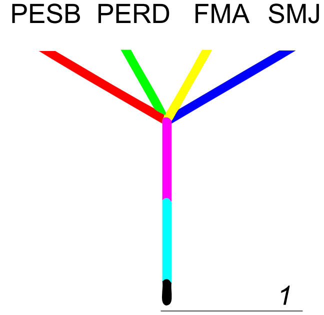

	Priors	Conditions
N1	10-10000	
N2	10-10000	
N3	10-10000	
N4	10-10000	
N1a	10000-100000	>N1,N2,N3,N4
N1b	10000-100000	>N1,N2,N3,N4
Nanc	10000-100000	>N1,N2,N3,N4

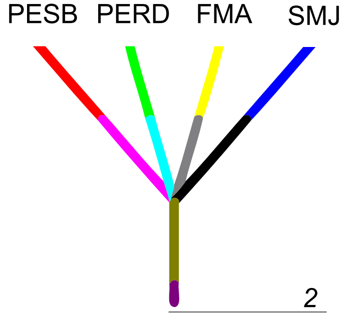

	Priors	Conditions
N1	10-10000	
N2	10-10000	
N3	10-10000	
N4	10-10000	
N2a	10-10000	>N1
N2b	10-10000	>N2
N2c	10-10000	>N3
N2d	10-10000	>N4
N2e	10000-100000	>N1,N2,N3,N4,2a,2b,2c,2d
Nanc	10000-100000	>N1,N2,N3,N4

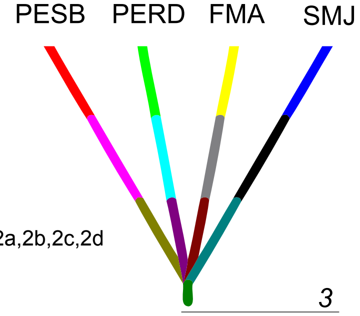

	Priors	Conditions
N1	10-10000	
N2	10-10000	
N3	10-10000	
N4	10-10000	
N3a	10-10000	>N1
N3b	10-10000	>N2
N3c	10-10000	>N3
N3d	10-10000	>N4
N3e	10-10000	
N3f	10-10000	
N3g	10-10000	
N3h	10-10000	
Nanc	10000-100000	>N1,N2,N3,N4, 3e,3f,3g,3h

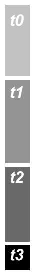

Sequential

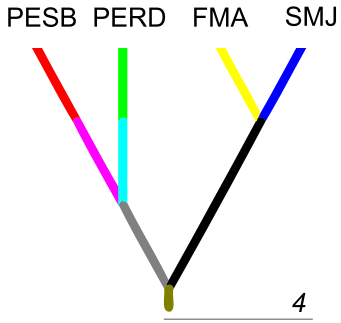

	Priors	Conditions
N1	10-10000	
N2	10-10000	
N3	10-10000	
N4	10-10000	
N4a	10-10000	>N1
N4b	10-10000	>N2
N4c	10-20000	>N3,N4
N4d	10-20000	>N4a,N4b
Nanc	10000-100000	>N1,N2,N3,N4,4c,4d

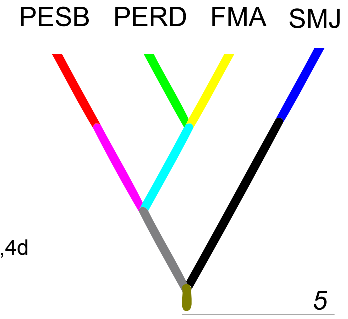

	Priors	Conditions
N1	10-10000	
N2	10-10000	
N3	10-10000	
N4	10-10000	
N5a	10-10000	>N1
N5b	10-10000	>N2,N3
N5c	10-20000	>N4
N5d	10-30000	>N5a,N5b
Nanc	10000-100000	>N1,N2,N3,N4,5c,5d

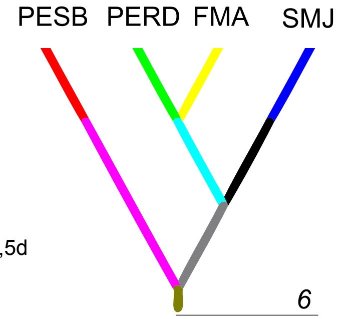

	Priors	Conditions
N1	10-10000	
N2	10-10000	
N3	10-10000	
N4	10-10000	
N6a	10-20000	>N1
N6b	10-20000	>N2,N3
N6c	10-10000	>N4
N6d	10-30000	>N6b,N5c
Nanc	10000-100000	>N1,N2,N3,N4,6a,6d

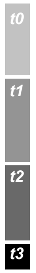

Sequential with admixture

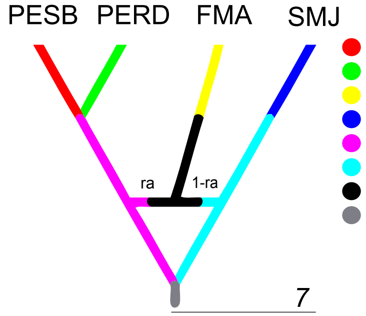

	Priors	Conditions
N1	10-10000	
N2	10-10000	
N3	10-10000	
N4	10-10000	
N7a	10-20000	>N1,N2
N7b	10-20000	>N4
N7c	10-10000	>N3
Nanc	10000-100000	>N1,N2,N3,N4,7a,7b
ra	0.001-0.999	

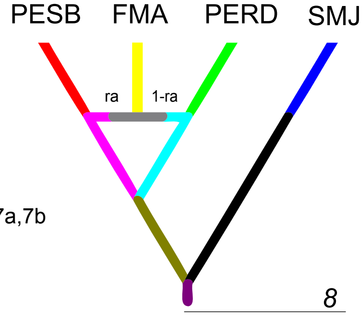

	Priors	Conditions
N1	10-10000	
N2	10-10000	
N3	10-10000	
N4	10-10000	
N8a	10-10000	>N1
N8b	10-10000	>N2
N8c	10-10000	>N3
N8d	10-20000	>N4
N8e	10-30000	>N8a,N8b
Nanc	10000-100000	>N1,N2,N3,N4,8d,8e
ra	0.001-0.999	

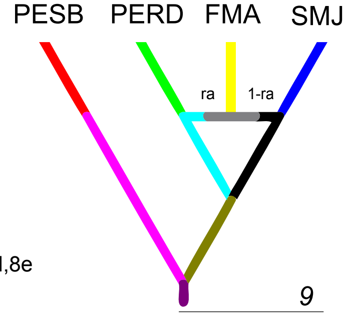

	Priors	Conditions
N1	10-10000	
N2	10-10000	
N3	10-10000	
N4	10-10000	
N9a	10-20000	>N1
N9b	10-10000	>N2
N9c	10-10000	>N3
N9d	10-10000	>N4
N9e	10-30000	>N9b,N9d
Nanc	10000-100000	>N1,N2,N3,N4,9a,9e
ra	0.001-0.999	

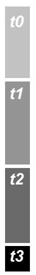

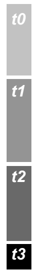

	Priors	Conditions
t0	present	
t1	1-1000	
t2	1-2000*	>t1
t3	1-30000	>t2

Summary statistics

**One sample**	# haplotypes<br># segregating sites<br>mean pairwise differences<br>Tajima's D
**Two sample**	# haplotypes<br># segregating sites<br>mean pairwise differences<br>Fst
**Admixture**	Samples FMA/PESB/PERD<br>Samples FMA/PERD/SMJ

Mutation model priors

	Min. - Max.
Mean μ (/s/generation)	1.0E-7 - 1.0E-6
Mean kappa	25 - 45
Model	HKY + I
% Inv. sites	90

* All Priors were Uniform, except for t2, which had a Normal distribution with mean 1500 and standard deviation 250.Time is in genenrations (~10 years/generation).

We followed the DIYABC manual to find appropriate prior model combinations for our data set. We used the same samples from the 106 samples data set to perform the ABC analysis though we included only the four populations shown on the diagrams above (N=90).
