# Supplementary material for: Genetic Diversity and Population History of a Critically Endangered Primate, the Northern Muriqui (Brachyteles hypoxanthus)
Source: PLoS One. 2011 Jun 3;6(6):e20722. doi: 10.1371/journal.pone.0020722 (PMC3108597; doi:10.1371/journal.pone.0020722)
Supplement: Table S1 — Absolute and relative frequencies of haplotypes per population (and group, if known). (DOC) [file pone.0020722.s002.doc]

**Table S1. Absolute and relative frequencies** of haplotypes per population (and group, if known).

| Haplotype | Minas Gerais (MG) state | | | | | | | | | Espírito Santo (ES) state | | | | | | | | | Overall |
| --- | --- | --- | --- | --- | --- | --- | --- | --- | --- | --- | --- | --- | --- | --- | --- | --- | --- | --- | --- |
|  | FE | RPPN  MS | PEI | PERD | PESB | RPPN-FMA | | | | PNC | | | SMJ | | | | | |  |
|  |  |  | ML | LP | MTP | M | J | M2 | N |  | VC | VSM | SSB | RP1 | RP2 | CO1 | CO2 | RCT |  |
| h1 |  |  |  |  | 6 |  |  |  |  |  |  |  |  |  |  | 3 |  |  | 9 |
| h2 |  |  |  | 1 | 2 |  |  |  |  |  | 1 |  |  |  |  |  |  |  | 4 |
| h3 | 2 |  |  | 7 |  |  |  |  |  |  |  |  |  | 1 | 1 | 1 | 1 |  | 13 |
| h4 |  | 11 |  | 1 |  |  |  |  |  |  |  | 1 |  |  |  |  |  |  | 13 |
| h5 |  |  | 1 |  |  |  |  |  |  |  |  |  |  |  |  |  |  |  | 1 |
| h6 |  |  |  | 6 |  |  |  |  |  |  |  |  |  |  |  |  |  |  | 6 |
| h7 |  |  |  | 1 |  |  |  |  |  |  |  |  |  |  |  |  |  |  | 1 |
| h8 |  |  |  | 5 |  |  |  |  |  |  |  |  |  |  |  |  |  |  | 5 |
| h9 |  |  |  | 1 |  |  |  |  |  |  |  |  |  |  |  |  |  |  | 1 |
| h10 |  |  |  | 1 |  |  |  |  |  |  |  |  |  |  |  |  |  |  | 1 |
| h11 |  |  |  |  | 2 |  |  |  |  |  |  |  |  |  |  |  |  |  | 2 |
| h12 |  |  |  |  | 3 |  |  |  |  |  |  |  |  |  |  |  |  |  | 3 |
| h13 |  |  |  |  | 2 |  |  |  |  |  |  |  |  |  |  |  |  |  | 2 |
| h14 |  |  |  |  | 1 |  |  |  |  |  |  |  |  |  |  |  |  |  | 1 |
| h15 |  |  |  |  | 1 |  |  |  |  |  |  |  |  |  |  |  |  |  | 1 |
| h16 |  |  |  |  |  | 11 | 6 | 4 | 12* |  |  |  |  |  |  |  |  |  | 33* |
| h17 |  |  |  |  |  | 11 | 5 | 1 |  |  |  |  |  |  |  |  |  |  | 17 |
| h18 |  |  |  |  |  | 9 | 3 |  | 2 |  |  |  |  |  |  |  |  |  | 14 |
| h19 |  |  |  |  |  |  |  |  |  |  |  |  |  |  |  | 6 |  | 1 | 7 |
| h20 |  |  |  |  |  |  |  |  |  |  |  |  | 14* |  |  |  |  |  | 14* |
| h21 |  |  |  |  |  |  |  |  |  |  |  |  |  | 1 |  |  |  | 1 | 2 |
| h22 |  |  |  |  |  |  |  |  |  |  |  |  |  | 1 |  |  |  |  | 1 |
| h23 |  |  |  |  |  |  |  |  |  |  |  |  |  |  |  | 1 |  |  | 1 |
| N° individuals | 2 | 11 | 1 | 23 | 17 | 31 | 14 | 5 | 14 |  | 1 | 1 | 14 | 3 | 1 | 11 | 1 | 2 | 152 |
| N° haplotype | 1 | 1 | 1 | 8 | 7 | 3 | 3 | 2 | 2 |  | 1 | 1 | 1 | 3 | 1 | 4 | 1 | 2 | 23 |
| N_hap_/N_ind_ (population) | 0.5 | 0.09 | 1.0 | 0.35 | 0.41 |  |  | 0.05 |  |  | 1.0 | | 0.28 | | | | | | 0.15 |

**P*<0.05 (randomization tests-of-goodness-of-fit P-value, see text for details)

**Population name: FE:** Fazenda Esmeralda, **RPPN-MS**: Reserva Particular do Patrimônio Natural Mata do Sossego, **PEI**: Parque Estadual do Ibitipoca, **PERD:** Parque Estadual do Rio

Doce, **PESB**: Parque Estadual da Serra do Brigadeiro, **RPPN-FMA:** Reserva Particular do Patrimônio Natural Feliciano Miguel Abdala, **PNC:** Parque Nacional do Caparaó**, SMJ:** Santa Maria

de Jetibá.

**Group name (size when available): LP:** Lagoa Preta; **MTP:** Matipó; **ML:** Mata do Luna (7); **M:** Matão (77), **J:** Jaó (53), **M2:** Matão 2 (37), **N:** Nadir (59); **SSB:** São Sebastião de Belém (16);

**RP1:** Rio das Pedras 1 (11); **RP2:** Rio das Pedras 2 (9); **CO1:** Córrego do Ouro 1 (11); **CO2:** Córrego do Ouro 2 (5); **RCT:** Rio Claro/Rio Triunfo (3); **VC:** Vale do Calçado (40); and **VSM:** Vale

Santa Marta.
