# Supplementary material for: Genetic Diversity and Population History of a Critically Endangered Primate, the Northern Muriqui (Brachyteles hypoxanthus)
Source: PLoS One. 2011 Jun 3;6(6):e20722. doi: 10.1371/journal.pone.0020722 (PMC3108597; doi:10.1371/journal.pone.0020722)
Supplement: Table S2 — Polymorphic sites for the HSVI segment in the northern muriqui. (DOC) [file pone.0020722.s003.doc]

**Table S2. Polymorphic sites for the HSVI segment in the northern muriqui.**

|  | **GenBank Accession Nos.** | **3** | **2**  **4** | ***6***  ***5*** | ***7***  ***4*** | **9**  **2** | ***1***  ***1***  ***9*** | ***1***  ***3***  ***9*** | **1**  **5**  **6** | ***1***  ***6***  ***8*** | ***1***  ***7***  ***4*** | **1**  **8**  **5** | ***1***  ***9***  ***1*** | ***1***  ***9***  ***2*** | ***2***  ***0***  ***4*** | ***2***  ***3***  ***2*** | ***2***  ***3***  ***5*** | **2**  **4**  **8** | ***2***  ***5***  ***1*** | ***2***  ***6***  ***1*** | **2**  **7**  **7** | ***2***  ***7***  ***8*** |
| --- | --- | --- | --- | --- | --- | --- | --- | --- | --- | --- | --- | --- | --- | --- | --- | --- | --- | --- | --- | --- | --- | --- |
| ***Reference*** | **AF213966** | **A** | **T** | **A** | **C** | **A** | **A** | **T** | **C** | **C** | **T** | **C** | **A** | **T** | **T** | **C** | **T** | **T** | **G** | **T** | **C** | **A** |
| **h1** | JF769864 | . | . | G | . | . | . | . | . | . | . | . | G | . | . | . | . | . | A | . | . | G |
| **h2** | JF769865 | . | . | . | . | . | . | C | . | . | . | . | . | C | C | . | . | . | A | . | . | . |
| **h3** | JF769866 | . | . | . | . | . | . | . | . | . | . | . | . | . | . | . | . | . | . | . | . | . |
| **h4** | JF769867 | . | . | . | T | . | . | . | . | . | . | . | . | . | . | . | C | . | . | . | . | . |
| **h5** | JF769868 | . | . | . | T | . | G | . | . | . | . | . | . | . | . | . | C | . | . | C | . | . |
| **h6** | JF769869 | . | . | G | . | . | . | . | . | . | . | . | . | C | C | . | . | . | A | . | . | . |
| **h7** | JF769870 | . | . | . | . | . | . | . | . | . | . | . | . | . | . | T | . | . | . | . | . | . |
| **h8** | JF769871 | . | . | . | T | . | . | . | . | . | . | . | . | . | . | . | C | . | . | C | . | . |
| **h9** | JF769872 | . | . | G | . | . | . | . | . | . | . | . | . | . | C | . | . | . | A | C | T | . |
| **h10** | JF769873 | . | . | G | . | G | . | . | . | T | . | . | G | . | . | . | . | . | A | . | . | G |
| **h11** | JF769874 | . | . | G | . | . | . | . | . | T | . | . | G | . | . | . | . | . | A | . | . | G |
| **h12** | JF769875 | . | . | . | T | . | . | . | . | . | . | . | . | C | . | . | C | . | . | C | . | . |
| **h13** | JF769876 | . | . | . | . | . | . | . | . | . | . | T | . | . | C | . | . | . | A | . | . | G |
| **h14** | JF769877 | . | . | . | . | . | . | . | . | . | . | . | . | . | . | . | . | . | A | . | . | G |
| **h15** | JF769878 | . | . | G | . | . | . | . | . | . | . | . | G | . | C | . | . | . | A | . | . | G |
| **h16** | JF769879 | G | . | . | . | . | . | C | . | T | . | . | G | . | . | . | . | . | A | . | . | G |
| **h17** | JF769880 | . | . | . | . | . | . | . | . | T | C | . | . | . | . | . | . | . | . | . | . | . |
| **h18** | JF769881 | . | . | . | . | . | . | . | . | T | C | . | . | . | . | . | . | C | . | . | . | . |
| **h19** | JF769882 | . | . | . | . | . | . | . | . | . | . | . | . | . | C | . | . | . | A | . | . | . |
| **h20** | JF769883 | . | . | . | T | . | . | . | . | . | . | . | . | C | . | . | C | . | . | . | . | . |
| **h21** | JF769884 | . | . | . | . | . | . | . | . | T | . | . | G | . | . | . | . | . | A | . | . | G |
| **h22** | JF769885 | . | A | . | . | . | . | . | . | T | . | . | G | . | . | . | . | . | A | . | . | G |
| **h23** | JF769886 | . | . | . | T | . | G | . | T | . | . | . | . | . | . | . | C | . | . | C | . | . |

Haplotypes (horizontal) and polymorphic sites (vertical) of northern muriqui HVSI. Vertical numbers indicates

a polymorphic position in the 366-bp alignment. Dots represent nucleotide identity between one particular

haplotype and the reference GenBank sequence (accession number AF213966). The T/A transversion in

haplotype h22 is in the 24th position. Parsimony informative sites are in italics.
