# Supplementary material for: Genetic Diversity and Population History of a Critically Endangered Primate, the Northern Muriqui (Brachyteles hypoxanthus)
Source: PLoS One. 2011 Jun 3;6(6):e20722. doi: 10.1371/journal.pone.0020722 (PMC3108597; doi:10.1371/journal.pone.0020722)
Supplement: Table S3 — Series of 16 AMOVAs excluding one group from both RPPN-FMA and SMJ at a time. (DOC) [file pone.0020722.s004.doc]

**Table S3. Series of 16 AMOVAs excluding one group from both RPPN-FMA and SMJ at a time.**

| RPPN-FMA group excluded | SMJ group excluded | Φ_SC_ | *P* |
| --- | --- | --- | --- |
| Nadir | SSB | 0.04 | **0.209*** |
|  | RP1/RP2 | 0.33 | <0.001 |
|  | CO1/CO2 | 0.29 | <0.001 |
|  | RCT | 0.34 | <0.001 |
| Matão | SSB | 0.13 | **0.057*** |
|  | RP1/RP2 | 0.45 | <0.001 |
|  | CO1/CO2 | 0.44 | <0.001 |
|  | RCT | 0.45 | <0.001 |
| Jaó | SSB | 0.23 | **0.003*** |
|  | RP1/RP2 | 0.45 | <0.001 |
|  | CO1/CO2 | 0.44 | <0.001 |
|  | RCT | 0.45 | <0.001 |
| Matão 2 | SSB | 0.15 | **0.009*** |
|  | RP1/RP2 | 0.37 | <0.001 |
|  | CO1/CO2 | 0.34 | <0.001 |
|  | RCT | 0.37 | <0.001 |
| No group excluded |  | 0.35 | <0.001 |

*Non-significant results after the serial goodness-of-fit plus correction (Carvajal-Rodríguez

& de Uña-Alvarez 2010).
