# Supplementary material for: Genetic Diversity and Population History of a Critically Endangered Primate, the Northern Muriqui (Brachyteles hypoxanthus)
Source: PLoS One. 2011 Jun 3;6(6):e20722. doi: 10.1371/journal.pone.0020722 (PMC3108597; doi:10.1371/journal.pone.0020722)
Supplement: Table S4 — Tamura-Nei pairwise genetic distances between haplotypes. [file pone.0020722.s005.doc]

**Table S4. Tamura-Nei pairwise genetic distances between haplotypes**.

|  | **h1** |  |  |  |  |  |  |  |  |  |  |  |  |  |  |  |  |  |  |  |  |  |
| --- | --- | --- | --- | --- | --- | --- | --- | --- | --- | --- | --- | --- | --- | --- | --- | --- | --- | --- | --- | --- | --- | --- |
| **h2** | 0.017 | **h2** |  |  |  |  |  |  |  |  |  |  |  |  |  |  |  |  |  |  |  |  |
| **h3** | 0.012 | 0.011 | **h3** |  |  |  |  |  |  |  |  |  |  |  |  |  |  |  |  |  |  |  |
| **h4** | 0.018 | 0.018 | 0.006 | **h4** |  |  |  |  |  |  |  |  |  |  |  |  |  |  |  |  |  |  |
| **h5** | 0.024 | 0.024 | 0.011 | 0.006 | **h5** |  |  |  |  |  |  |  |  |  |  |  |  |  |  |  |  |  |
| **h6** | 0.011 | 0.006 | 0.011 | 0.017 | 0.024 | **h6** |  |  |  |  |  |  |  |  |  |  |  |  |  |  |  |  |
| **h7** | 0.015 | 0.014 | 0.003 | 0.009 | 0.014 | 0.014 | **h7** |  |  |  |  |  |  |  |  |  |  |  |  |  |  |  |
| **h8** | 0.021 | 0.021 | 0.009 | 0.003 | 0.003 | 0.021 | 0.012 | **h8** |  |  |  |  |  |  |  |  |  |  |  |  |  |  |
| **h9** | 0.014 | 0.014 | 0.014 | 0.021 | 0.020 | 0.009 | 0.017 | 0.017 | **h9** |  |  |  |  |  |  |  |  |  |  |  |  |  |
| **h10** | 0.006 | 0.024 | 0.018 | 0.024 | 0.030 | 0.017 | 0.021 | 0.027 | 0.020 | **h10** |  |  |  |  |  |  |  |  |  |  |  |  |
| **h11** | 0.003 | 0.020 | 0.015 | 0.021 | 0.027 | 0.014 | 0.018 | 0.024 | 0.017 | 0.003 | **h11** |  |  |  |  |  |  |  |  |  |  |  |
| **h12** | 0.024 | 0.018 | 0.012 | 0.006 | 0.006 | 0.017 | 0.015 | 0.003 | 0.021 | 0.030 | 0.027 | **h12** |  |  |  |  |  |  |  |  |  |  |
| **h13** | 0.011 | 0.011 | 0.011 | 0.017 | 0.024 | 0.011 | 0.014 | 0.021 | 0.014 | 0.017 | 0.014 | 0.024 | **h13** |  |  |  |  |  |  |  |  |  |
| **h14** | 0.006 | 0.012 | 0.006 | 0.011 | 0.018 | 0.011 | 0.009 | 0.014 | 0.014 | 0.012 | 0.009 | 0.018 | 0.006 | **h14** |  |  |  |  |  |  |  |  |
| **h15** | 0.003 | 0.014 | 0.015 | 0.021 | 0.027 | 0.009 | 0.018 | 0.024 | 0.011 | 0.008 | 0.006 | 0.027 | 0.009 | 0.009 | **h15** |  |  |  |  |  |  |  |
| **h16** | 0.011 | 0.017 | 0.018 | 0.024 | 0.030 | 0.024 | 0.021 | 0.027 | 0.027 | 0.012 | 0.008 | 0.030 | 0.017 | 0.011 | 0.014 | **h16** |  |  |  |  |  |  |
| **h17** | 0.018 | 0.018 | 0.006 | 0.012 | 0.018 | 0.017 | 0.009 | 0.015 | 0.021 | 0.018 | 0.015 | 0.018 | 0.017 | 0.011 | 0.021 | 0.018 | **h17** |  |  |  |  |  |
| **h18** | 0.021 | 0.021 | 0.009 | 0.015 | 0.021 | 0.021 | 0.012 | 0.018 | 0.024 | 0.021 | 0.018 | 0.022 | 0.021 | 0.014 | 0.024 | 0.021 | 0.003 | **h18** |  |  |  |  |
| **h19** | 0.012 | 0.006 | 0.006 | 0.011 | 0.017 | 0.006 | 0.008 | 0.014 | 0.008 | 0.018 | 0.014 | 0.018 | 0.006 | 0.006 | 0.009 | 0.017 | 0.011 | 0.014 | **h19** |  |  |  |
| **h20** | 0.021 | 0.014 | 0.009 | 0.003 | 0.008 | 0.014 | 0.012 | 0.006 | 0.024 | 0.027 | 0.024 | 0.003 | 0.021 | 0.014 | 0.024 | 0.027 | 0.015 | 0.018 | 0.014 | **h20** |  |  |
| **h21** | 0.006 | 0.017 | 0.012 | 0.017 | 0.024 | 0.017 | 0.014 | 0.021 | 0.021 | 0.006 | 0.003 | 0.024 | 0.011 | 0.006 | 0.008 | 0.006 | 0.012 | 0.014 | 0.011 | 0.021 | **h21** |  |
| **h22** | 0.008 | 0.020 | 0.014 | 0.020 | 0.027 | 0.020 | 0.017 | 0.023 | 0.023 | 0.008 | 0.006 | 0.027 | 0.014 | 0.008 | 0.011 | 0.008 | 0.014 | 0.017 | 0.014 | 0.023 | 0.003 | **h22** |
| **h23** | 0.027 | 0.027 | 0.014 | 0.008 | 0.003 | 0.027 | 0.018 | 0.006 | 0.024 | 0.034 | 0.030 | 0.008 | 0.027 | 0.021 | 0.030 | 0.033 | 0.021 | 0.024 | 0.021 | 0.011 | 0.027 | 0.030 |
