# Supplementary material for: Genetic Diversity and Population History of a Critically Endangered Primate, the Northern Muriqui (Brachyteles hypoxanthus)
Source: PLoS One. 2011 Jun 3;6(6):e20722. doi: 10.1371/journal.pone.0020722 (PMC3108597; doi:10.1371/journal.pone.0020722)
Supplement: Table S5 — Summary table of genetic diversity parameters of HVSI sequences in northern muriquis and available neotropical primates. (DOC) [file pone.0020722.s006.doc]

**Table S5. Summary table of genetic diversity parameters of HVSI sequences in northern muriquis and available neotropical primates.**

| Species | IUCN^1^ | N | Sites | Haplotypes | Base pairs | π | *h* | Reference |
| --- | --- | --- | --- | --- | --- | --- | --- | --- |
| *Brachyteles hypoxanthus* | CR | 152 | 8 | 23 | 366 | 0.0135 | 0.905 | This study |
| *Alouatta caraya* | LC | 73 | 6 | 31 | 370 | 0.0149 | 0.933 | Ascunce *et al*. 2007 |
| *Ateles belzebuth* | EN | 11 | 9 | 11 | 354 | 0.0816 | 1.000 | Collins & Dubach 2000 |
| *Callithrix jacchus* | LC | 77 | 4 | 22 | 363 | 0.0247 | 0.895 | Faulkes *et al*. 2003 |
| *Lagothrix lagotricha* | VU | 61 | 3 | 31 | 366 | 0.0357 | 0.970 | Di Fiore 2009 |
| *Saguinus mystax* | LC | 69 | 1 | 14 | 376 | 0.0135 | 0.800 | Huck *et al*. 2007 |
| *Saguinus niger* | VU | 22 | 3 | 21^2^ | 361 | 0.0477 | 0.987 | Vallinoto *et al*. 2006 |

^1^IUCN redlist category – LC: Least Concern, VU: Vulnerable, EN: Endangered, CR: Critically Endangered

^2^Sequence "XE8" was inferred from the phylogenetic tree

Ascunce MS, Hasson E, Mulligan CJ, Mudry MD (2007) Mitochondrial sequence diversity of the southernmost extant New World monkey, *Alouatta caraya*. Mol Phylogen Evol 43: 202-215.

Collins AC, Dubach JM (2000) Phylogenetic relationships of spider monkeys (*Ateles*) based on mitochondrial DNA variation. Int J Primatol 21: 381-420.

Di Fiore A (2009) Genetic approaches to the study of dispersal and kinship in New World primates. In: Garber PA, Estrada A, Bicca-Marques JC, Heymann EW, Strier KB, editors. South American primates: comparative perspectives in the study of behavior, ecology, and conservation. New York: Springer Science+Business Media. pp. 211-250.

Faulkes, CG, Arruda, MF, & Da Cruz AOM 2003, 'Matrilineal genetic structure within and among populations of the cooperatively breeding common marmoset, *Callithrix jacchus*', *Molecular Ecology* vol. 12, no. 4, April, pp 1101-1108.

Huck M, Roos C, Heymann EW (2007) Spatio-genetic population structure in mustached tarnarins, *Saguinus mystax*. Am J Phys Anthropol 132: 576-583.

Vallinoto M, Araripe J, do Rego PS, Tagliaro CH, Sampaio I, et al. (2006) Tocantins river as an effective barrier to gene flow in *Saguinus niger* populations. Genet Mol Biol 29: 215-219.
